# Neuropsychological Tests in Post-operative Cognitive Dysfunction: Methods and Applications

**DOI:** 10.3389/fpsyg.2021.684307

**Published:** 2021-06-04

**Authors:** Jun Liu, Kequn Huang, Binbin Zhu, Bin Zhou, Ahmad Khaled Ahmad Harb, Lin Liu, Xiang Wu

**Affiliations:** Department of Anesthesiology, The Affiliated Hospital of Medical School, Ningbo University, Ningbo, China

**Keywords:** anesthesia, surgery, neuropsychological assessment, cognitive dysfunction, perioperative

## Abstract

Post-operative cognitive dysfunction (POCD) is a neurological complication that relatively frequently occurs in older people after anesthesia/surgery, with varying durations and significant differences in the severity of cognitive impairment. POCD is mainly characterized by memory loss mostly without consciousness disorders, accompanied by abnormal emotions, behaviors, and language, mostly without consciousness disorder. The clinical performance of POCD lacks specificity but can reflect the severity of cognitive impairment in patients. The diagnosis of POCD cannot be separated from the evaluation of perioperative cognitive function of patients, and the more popular and accepted method is neuropsychological tests (NPTs).

Post-operative cognitive dysfunction (POCD) is the functional impairment of the nervous system activities, such as memory, executive function, language, direction, emotion, visual space structural ability, in patients after surgery/anesthesia, which is a kind of reversible mild cognitive impairment (MCI). Most of them last for a short period (Abildstrom et al., 2000). POCD can hinder patients from recovery, prolong the length of stay, as well as increase mortality (Monk et al., 2008), and the risk of leaving the labor market prematurely (Steinmetz et al., 2009). In 2018, several studies (Evered et al., 2018) recommended perioperative neurocognitive disorders (PNDs) to replace POCD. PND facilitates diagnosing cognitive changes after anesthesia/surgery with the diagnostic criteria of neurocognitive disorder in the general population. Neuropsychological tests (NPTs) are often used to assess cognitive function in perioperative patients. The timing of neuropsychological evaluation of patients and the specificity and sensitivity of NPTs may all have an impact on the diagnosis of POCD. There are many kinds of NPTs for evaluation, but there is no uniform and complete set of NPT batteries as the diagnostic criteria for POCD. In this review, we highlight the common NPTs of POCD and their development.

## Post-operative Cognitive Dysfunction and Post-operative Delirium

Post-operative delirium (POD) and POCD are common complications after surgery. They are two different pathological states but with some correlation. POD is an acute and severe cognitive impairment that mostly occurs in 1–3 days after surgery. POCD is more likely to appear 1 week after surgery and last for several months and even longer (Newman et al., 2001; Pérez-Belmonte et al., 2019). There is a specific diagnosis criterion for POD but POCD does not have a recognized one. However, after the recommendation of nomenclature of perioperative cognitive change, POD and POCD are integrated into PNDs and obtain their new and specific definitions and criteria (Evered et al., 2018; Daiello et al., 2019). They share some common risk factors in which age and the level of education are independent and crucial (Monk et al., 2008). But the diminished “cognitive reserve” may be the real reason hidden behind them. POD is related to cognitive dysfunction in a short period after surgery (Sauër et al., 2017; Daiello et al., 2019). At the same time, delirium is likely to interface with dementia on multiple levels, and POD implies a decline in cognitive reserve, predicting a decline in cognitive function and dementia in the future (Nadelson et al., 2014; Fong et al., 2015). Preoperative cognitive impairment such as MCI and early dementia will facilitate the occurrence of POCD (Kline et al., 2012). It has been proposed that POCD may be associated with the occurrence and development of post-operative dementia, based on that anesthesia can lead to the oligomerization and deposition of amyloid β-protein (Fodale et al., 2010), which can promote the pathological pathway of dementia. However, according to an 11-year prospective study, there is no significant correlation between POCD and the occurrence of dementia (Steinmetz et al., 2013). So the relationship between post-operative complications and dementia is still unclear and further exploration will be needed to unmask it.

## Evaluation Development of POCD

In 1955, Bedford (1995) first reported a series of neurological complications, such as impaired directional function and forgetfulness of friends and household, in the elderly who had no abnormalities before surgery. Since then, more and more researchers have gradually paid more attention to it, but overall, research has been slow for a long time due to the lack of targeted assessment tools. Later, Moller et al. (1993) took patients with general anesthesia and local anesthesia as the same subjects, which may influence the incidence of POCD (Rasmussen et al., 2003). He also evaluated preoperative and post-operative cognitive function through the Wechsler Memory Scale (WMS) and continuous reaction time (RT) tests and used questionnaires to assess long-term cognitive function after surgery. Willner and Struve (1970) and Willner et al. (1976) used psycho-interviews and conceptual-level analogy test to evaluate preoperative and post-operative cognitive functions. Mattlar et al. (1991) used evaluation scales and NPTs for cognitive evaluation and used different test combinations at different stages. Chung et al. (1989) applied mini-mental state examination (MMSE) to assess cognitive function 6 h after surgery and 24 h after surgery. Murkin et al. (1995) carried out perioperative NPT and established a healthy control group. Cutoffs for “abnormal” functioning were operationally defined as change scores that were exceeded by 95% of the normative control group, and cognitive dysfunction was defined as impaired performance within one or more of the tests. Due to the limitations of the research methods at this stage, there were great differences among the results derived from all of the researchers in this field at this stage (Rasmussen et al., 2001).

In 1998, Moller et al. conducted a large-scale prospective study in which they used a rigorously screened NPT battery including visual–verbal learning test, concept shifting test, Stroop color–word interference test, pen and paper memory scanning test, letter-digital coding, and four-box test to evaluate the cognitive status and took Z-scores as the criterion of POCD (Moller et al., 1998). After that, other sets of NPT batteries derived from the previously mentioned tests began to be widely used for clinical evaluation. Clinical researchers are increasingly accepting the NPT-based Z-scores as the diagnosis of POCD. With the development of modern science and technology, cognitive evaluation assessment has evolved from traditional pen-and-paper tools to computer-based tests and then to utilizing lightweight and convenient tablet devices, which come with a digital set of assessment tools matched with it. Theoretically, the more NPTs there are in a battery, the more comprehensive and sensitive the evaluation of cognitive function is. Simultaneously, the testing time will increase accordingly, and the compliance of patient will be poor. Multiple NPTs exist with overlapping cognitive domains among them. Thus, NPTs with fewer overlapping portions that offer basic coverage of cognitive domains should be chosen to shorten the test time. Table 1 presents the batteries of NPTs used in clinical studies.

**Table 1:** NPT batteries in clinical studies.

**Author**	**Type of surgery/disease**	**Test battery**	**The diagnostic method**
Klinger et al. (2019)	Cardiac Surgery	Hopkins Verbal Learning Test, Randt Short Story Memory Test, Modified Visual Reproduction Test, Digit Span Test, Digit Symbol Test, Trail Making Test (part A, part B).	SD method
Sauër et al. (2017)	Cardiac Surgery	Coris Block-Tapping Task, Rey Auditory Verbal Learning Test, Grooved Pegboard Test, Trail Making Test (part A, part B), Digit Span Test (forward and backward).	Z-scores
Vedel et al. (2019)	Cardiac Surgery	Visual Verbal Learning Test, Concept Shifting Test, Stroop Color-Word Interference Test, Letter Digit Coding Test.	Z-scores
Quan et al. (2019)	Non-Cardiac Surgery	Mental Control Digit Span Test (forward and backward), Visual Retention Test, Paired Associate Verbal Learning Test, Digit Symbol Test, Trail Making Test (part A), Grooved Pegboard Test (favored and unfavored hand).	SD method
Deng et al. (2021)	Non-Cardiac Surgery	Rey Auditory Verbal Learning Test, Stroop Color-Word Interference Test, Trail Making Test (part A, part B), Digit Span Test (forward and backward), Digit Symbol Test, Purdue Pegboard Test (dominant and non-dominant hand), Verbal Fluency Test.	SD method
Berger et al. (2019)	Non-Cardiac Surgery	Randt Short Story Test, WMS Modified Visual Reproduction Test, Digit Span Test, Digit Symbol Test, Trail Making Test (part B), Rey Auditory Verbal Learning Test.	SD method
Langer et al. (2019)	Non-Cardiac Surgery	Trail Making Test (part A, part B), Stroop Color-Word Interference Test, Digit Symbol Test, Free, and Cued Selective Reminding Test, Verbal Phonemic Fluency Test, Denomination Test.	Z-scores
Deiner et al. (2019)	Non-Cardiac Surgery	Trail Making Test (part A, part B), Logical Memory Test, California Verbal Learning Test, Verbal Fluency Test (animals and plants), Digit Span Test (forward and backward), Digit Symbol Test, Boston naming Test.	SD method
Li et al. (2019)	Total Hip/Knee Replacement Surgery	MoCA, Stroop Color-Word Interference Test, Digit Span Test, Digit Symbol Test, Associative Learning, and Memory Test.	Z-scores
Silbert et al. (2015)	Hip Replacement Surgery	Auditory verbal Learning Test, Trail Making Test (part A, part B), Digit Symbol Test, Controlled Oral Word Association Test, Semantic fluency test (animals), Grooved Pegboard Test (favored and unfavored hand).	Z-scores
Tanaka et al. (2017)	Full Knee Replacement	Digit Symbol Test, Trail Making Test, Digit Span Test.	Percentage method (20%)
Geng et al. (2017)	Laparoscopic Cholecystectomy	Vision test, Digit Symbol Test, Cumulative Test, Digit Span Test (forward and backward), Trail Making Test (part A), auditory language learning test, groove nail plate test.	Percentage method (20%)
Hoffmann et al. (2009)	Stroke	Coconuts test	SD method

## Common NPTS and Their Characteristics

### A List of Primary Screening Tests Commonly Used for POCD

Clinically, MMSE and Montreal Cognitive Assessment Scale (MoCA) are used for the initial screening of patients, but these tools are not suitable for follow-up evaluation of patients.

#### Mini-Mental State Examination

Mini-mental state examination is currently the scale of evaluating cognitive function widely used in the clinical environment. It includes time and spatial orientation, language ability, instant memory, delayed recall, attention, calculation, visual–spatial ability, and executive function. The scale is simple and easy to conduct, which is suitable for the evaluation of elderly patients. The characteristic of covering the majority of cognitive domains makes it suitable for screening cognitive function in the population. However, every coin has two sides. Consequently, it demonstrates low sensitivity and the ceiling effect when this scale is used to detect impairment of a single cognitive domain and mild changes. The total points are 30, and 23/24 is the original cutoff score (Folstein et al., 1975), but the corresponding sensitivity is low. As the cutoff value increases, the sensitivity gradually ascends, and the specificity gradually descends. Taking 26/27 as the boundary point can attain proper sensitivity and specificity.

#### Montreal Cognitive Assessment Scale

Nasreddine et al. developed MoCA in Montreal, Canada, in 1995, for health professionals to assess mild cognitive dysfunction. This scale evaluates cognitive function from visuospatial, executive function, naming, memory, attention, language, abstraction, and orientation. Furthermore, it is commonly used for screening MCI. A score of 24/25 out of 30 is recommended as the cutoff score for MCI (sensitivity: 80.48%, specificity: 81.19%), and MoCA is more suitable for screening MCI compared with MMSE (Nasreddine et al., 2005; Ciesielska et al., 2016).

### NPTs Are Commonly Used to Evaluate Cognitive Status

#### Visual/Auditory Verbal Learning Test

The visual/auditory verbal learning test is a task of verbal memory designed to measure the ability to learn new verbal information and retrieve it from memory. In other words, the test is mainly to assess working memory. Specialists visually display through a booklet or screen or read 15 low-associated words. This process is repeated five times. After each process, the patient is asked to recall as many words as possible, and after 20 min of the last presentation or reading, the subject is told to recall as many words as possible (Brand and Jolles, 1985). The verbal memory test is particularly sensitive to the early stages of cognitive impairment, and can effectively distinguish between MCI and normal condition. The test is simple and easy to practice. Using two different but equivalent sets of words during preoperative and post-operative assessments can alleviate learning effects. The visual-verbal learning test is also a subtest of the recommended test battery for the International Study of Post-operative Cognitive Dysfunction (ISPOCD). In the literature, it is reported that the telephone version of the Rey Auditory Verbal Learning Test (RAVL) has demonstrated good known-groups effectiveness in distinguishing subjective cognitive impairment and amnestic MCI (aMCI) and is significantly correlated with the regular Hopkins Verbal Learning Test (Jagtap et al., 2020). The verbal learning test could be influenced by many factors, such as the speed of presentation, level of education, age, gender, vision, and hearing.

#### Stroop Color Interference Test

The test was originally developed by Stroop in 1935. It is used to evaluate selective attention, information processing speed, execution, and anti-interference ability. This initial version is composed of three white cards with every card containing 50 items. This test includes four parts: in part 1, the subject is asked to read the randomized color names (green, blue, red, brown, and purple) printed in black. For part 2, the subject needs to read the same color names as part 1 printed in incongruously colored ink (blue, green, yellow, and red). Next in part 3, the subject has to name the same number of correspondingly colored patches. Finally, the subject should name the color of the ink printed in the card used in part 2 disregarding the color name. The time consumed during each part is recorded as scores (Houx et al., 1993). A lot of other versions are developed based on it and these tests are different in the color and the number of items. Stroop word-reading reflected the speed of visual search. Stroop color-naming reflected working memory as well as the speed of visual search. Stroop color-word reflected working memory, conflict monitoring, and speed of visual search (Periáñez et al., 2021). Because the test mainly reflects the attention of the subject, it is also often used for cognitive evaluation in patients with multiple sclerosis or schizophrenia, which can affect selective attention (Morrow, 2013). There are also some limitations of it: (1) Because the test is mainly carried out visually, patients with vision impairment or red–green colorblindness will not complete the test or get inaccurate test scores. (2) For illiterate patients, the third part of the test will not be able to effectively assess the selective attention of the patient, which is prone to a false negative. Some researchers recommend taking the numeral version of the Stroop test as an alternative to the Stroop color interference test (Kulaif and Valle, 2008), but its effectiveness still needs a great deal of research and studies to confirm.

#### Concept Shifting Test

A kind of NPT based on the trail making test (TMT) to assess the conceptual transfer and executive function in a patient. This test consists of four basic parts. The test material is a paper with four large circles, in which 16 small circles are grouped in a larger circle. The four large circles are assigned in random order with digits, letters, both digits and letters, and with nothing. The participants have to cross out the digits in numerical order, the letters in alphabetical order, the digits and letters in alternating order, and the empty circle in a clockwise orientation. Concept shifting test is usually used in common with Stroop test to perform executive evaluations, but it is reported that the test has low test–retest reliability (Rasmussen and Siersma, 2004). The test is susceptible to gender, age, and education level, while the preferred hand does not affect test performance. In clinical and research domains, the scores participants acquired from this test will be compared to normal data to evaluate cognitive function.

#### Paper and Pencil Memory Screening Test

The test is improved based on the Sternberg memory screening test. It can evaluate working memory, visual scanning speed, and information-processing speed. Compared with the digit symbol test, it focuses more on the evaluation of working memory. In this test, the patient is first given a blank paper containing one to four letters, and after 5 s, it is replaced with a rectangular test sheet containing 120 random letters. The subject is asked to delete the previous four letters. The tools composing this test are simple, and the test time is short, ~5 min, taking the consumed time as the measurement is simpler than the scoring system of the original screening test. But the test also has some limitations of its own. For example, it is not suitable for subjects who are not familiar with the letters, and the test is also easily affected by age and education level (Van Der Elst et al., 2007).

#### Grooved Pegboard Test (GPT)

Grooved pegboard test can be used to evaluate the manual flexibility, fast visual–motor coordination, and psychomotor speed in the patient. The assessment tool is a metal plate composed of an array of 25 holes with differently positioned slots and pegs with a little ridge on the side. Only when the ridge is in the same orientation of the slot can the peg be inserted. The subject needs to insert these pegs into the holes in order as quickly as possible until all the holes have been filled, first with the dominant hand and then the non-dominant one. The conductor records the required time of each hand as the two parts of the scoring system. Studies have pointed out that the test has good test–retest reliability from 7 days to 3 months after surgery. This test can also be used for motor function in patients with Parkinson's disease and can adequately reflect the level of clinical assessment of upper-limb control, stiffness, and motor retardation (Sage et al., 2012). Women complete it faster than men. The higher the level of education is, the better the performance is, and the preferred hand has a better performance in contrast with the other hand. GPT also has a new version (removing task) corresponding to the standard version (placement task), and the new version is more susceptible to gender and use of preferred hand effects (Bryden and Roy, 2005). Adding cognitive or motor tasks to the GPT process can better assess manual flexibility and cognitive ability (Espenes et al., 2020).

#### Trail Making Test

Trail making test is one of the most widely used NPTs. This test includes two parts. Each part consists of 25 circles on a sheet of paper. The circle of TMT part A (TMT-A) contains consecutive numbers from 1 to 25, and participants need to connect them as quickly as possible in numerical order by making pencil lines. The circle of TMT part B (TMT-B) contains consecutive numbers from 1 to 13 and consecutive letters from A to L, and subjects need to connect numbers and letters in alternating order. The test yields two scores that are the needed time of these two parts. TMT-A provides a measurement of visual-scanning speed, while TMT-B additionally involves assessment of executive function. The score difference between TMT-B and TMT-A (TMT B-A) and the ratio of the two scores (TMT B/A) enhance the measurement of executive function. TMT is sensitive to a variety of diseases with neurological deficits; therefore, TMT is suitable as a screening tool for the evaluation of nervous system integrity and individual cognitive function. TMT would be easily affected by age and education level. Education level has a greater correlation with TMT-B. Correspondingly, its derived scores (TMT B-A and TMT B/A) are less affected by age and education level (Cavaco et al., 2013). Many people are not familiar with letters, so the original TMT-B would not be a fitting tool. To solve this problem, researchers developed some other versions, such as the version for Chinese (Lu and Bigler, 2000), but the corresponding norm data and the validity require additional research for further clarification.

#### Boston Naming Test (BNT)

Boston naming test is a subset of the Boston Diagnostic Aphasia Examination (BDAE). It is an extensive object-naming test that can be used to evaluate the ability of the patient to find and name words. The standard version of BNT (BNT-S) consists of 60 line drawings of common objects arranged in descending order of word frequency. Participants are asked to verbally speak out the name of the drawings, and some semantic or phonemic cues can be given to subjects when they are stuck in the test process. The scores consist of the number of correct items without cueing, the number of cues given, and the number of correct responses with each kind of cue. At the same time, the short versions of BNT containing 15 or 30 items were derived from it to reduce test time for patients. It is reported that the short BNT has a strong correlation with the BNT-S in outpatient evaluation (Attridge et al., 2020). All brief BNTs retain the function to distinguish between normal subjects and dementia subjects but compared with the full BNT, the short versions are weaker in distinguishing between normal and MCI subjects (Katsumata et al., 2015).

### A Complete Set of Cognitive Testing Tools

#### National Institutes of Health (NIH) Examiner

Executive function is an important part of cognitive assessment, but there is no consensus on the testing methods and operating procedures of executive function. Therefore, The National Institute of Neurological Disorders and Stroke had developed an NPT suited for executive function. This test group involves multiple areas associated with executive function: working memory, inhibition, scene switching, fluency, planning, insight, social cognition, and behavior, combining the scores of the subsets to form an executive composite score using the item response theory. The test group is modular, modifiable, efficient, and suitable for a wide range of age groups and cognitive levels. It has English and Spanish versions and includes computer-based and traditional paper-and-pencil testing formats (Kramer et al., 2014).

#### NIH Toolbox-Cognitive Battery

National Institutes of Health Toolbox is a comprehensive set of neuro-behavioral measurements, of which cognitive battery is the cognitive assessment part. The assessing process is based on a tablet computer and is simple and easy to implement. The battery consists of tests of seven constructs, including five fluid cognition (executive function, attention, episodic memory, working memory, and processing speed) and two crystallized cognition (vocabulary and word reading). This test group can maintain its stability among people of different age groups, races, genders, and education levels (Ma et al., 2020). It also has good reliability and validity in the evaluation of the healthy elderly (Scott et al., 2019). The decrease of the high-scores rate of the battery reflects the decline of cognitive function in participants with greater education and crystallized ability (Karr and Iverson, 2020). There are some other computer-based NPTs listed in Table 2.

**Table 2:** Computer-based NPTs.

**Computer-based tests**	**Components**	**Cognitive domains**	**Application way**	**Application time**
CNS Vital Signs(www.cnsvs.com)	Verbal Memory, Visual Memory, Finger Tapping, Symbol Digit Coding, Stroop Test, Shifting Attention, Continuous Performance, Perception of Emotions, Non-Verbal Reasoning, 4-Part Continuous Performance.	Composite memory, verbal memory, visual memory, working memory psychomotor speed, reaction time, complex attention, simple attention, sustained attention, cognitive flexibility, processing speed, motor speed, executive function, social acuity, reasoning.	Local software, web-based	–
King Devick Test (Rizzo et al., 2016)	Number Naming Test	Attention, processing speed, visual scanning	Local software	1–2 min
NeuroTrax—Mindstreams (Press et al., 2021)	Verbal Memory, Non-verbal memory, Go-NoGo, Stroop Test, Verbal Function, Problem Solving, Visual Spatial Imagery, Staged Information Processing, Finger Tapping, Catch Game.	Memory, executive function, visual spatial skills, verbal fluency, attention, information processing, motor skills.	Local software	About 45 min
ANAM GNS (Woodhouse et al., 2013)	Simple Reaction Time, Code Substitution Learning, Procedural Reaction Time, Mathematical Processing, Match to Sample, Code Substitution Delayed Memory	Motor speed, attention, learning, processing speed, visual scanning, basic computation, working memory, visuospatial discrimination, visual recognition, cognitive fatigue.	Local software	About 45 min
Cognivue (Cahn-Hidalgo et al., 2020)	Adaptive Motor Control Test, Visual Salience Test, Letter Discrimination, word Discrimination, Shape Discrimination, Motion Discrimination, Letter Memory, Word Memory, Shape Memory, Motion Memory.	Basic motor and visual ability, perceptual processing, memory processing.	Local software	10 min
CANTAB (Backx et al., 2020)	Reaction Time, Rapid Visual Information Processing, Motor Screening Task, Match to Sample Visual Search, Cambridge, Gambling Task, Multitasking Test, One Touch Stocking of Cambridge, Spatial working memory, Stop Signal Task, Delayed Matching to Samples, Paired Association Learning, Pattern Recognition Memory, Spatial Span, Verbal Recognition Memory, Emotional Bias Task, Emotional Recognition Task.	Attention and psychomotor sped, executive function, memory, emotion, and social cognition.	Local software, web-based	–
CANS-MCI (Memória et al., 2014)	General Reaction Time, Word-to-Picture Matching Latency, Design Matching, Stroop, Clock Hand Placement, Free Recognition, Guided Recognition, Picture Naming.	Executive function, memory, language fluency.	Local software	30–50 min
CogSport (Goh et al., 2021)	Detection Task, Identification Task, One Card Learning Task, One Back Task.	Processing speed, attention/vigilance, visual learning, executive function.	Local software	10–15 min
Headminder (Baker et al., 2018)	Number Recall, Number Sequencing, Memory Cabinet 2, Incidental Learning 1, Memory Cabinet 1, Incidental Learning 2, Response Direction 1, Response Direction 2, Animal Decoding, Symbol Scanning.	Attention, memory, processing speed, response speed.	Web-based	25–35 min

### Modern NPTs Based on the Internet and Artificial Intelligence

An online application (Lunardini et al., 2020) based on a tablet has attracted more attention, and its built-in voice assistant can realize fully autonomous evaluation. The NPT based on video conference can realize remote evaluation, which is of great significance for people in remote areas, but there are some limitations, for example, it cannot provide an evaluation of cognitive functions in comparison to hands-on tests. At the same time, whether video-related factors will impact on reliability and validity of the test have not been fully confirmed. Some initial evidence (Brearly et al., 2017) demonstrates the NPT mediated by language is less affected by video-related factors and particularly suitable as video-related subsets. Web-based evaluation methods are conducive to recruiting subjects and large-scale data collection and will be a potential development direction of cognitive evaluation in the future. Researchers are trying to use biomedical informatics for managing big data from advanced assessment methods, which can make more effective use of data sources (Miller, 2019).

With the advent of the era of intelligence, artificial intelligence has gradually been applied to the field of cognitive assessment. Kang et al. (2019) used artificial network algorithms to predict the impairment of cognitive function by summarizing multi-center NPT data, and both its predictive sensitivity and specificity were above 95%. Artificial intelligence is also applied in the field of Alzheimer's disease (AD) and has become a more powerful method of extracting reliable predictors and automatically classifying different phenotypes (Battista et al., 2020). The traditional cognitive-evaluation process is easily influenced by changes in the surrounding environment, and Virtual Reality (VR) technology can provide an immersive feeling as well as keep other interference relatively constant at each testing stage. Because VR has many advantages over traditional NPTs, investigators are paying more and more attention to the application of VR for cognitive testing and making neuropsychological decisions. Amato et al. (2020) used the NPT battery “CONVIRT,” fitted with VR for the evaluation of cognitive dysfunction caused by alcohol, which showed high sensitivity and test–retest reliability.

Although these NPTs can more accurately reflect the domain-specific change and severity of cognitive impairment, in the real clinical environment, the natural decline in the functional state in patients, or emotional and physical conditions influenced by surgery and disease itself, will lead to the patients not being in a suitable state to conduct NPT (Hoffmann, 2020). For example, patients with decreased concentration levels or abulia may not be fit for the relatively complex and time-consuming batteries, so the relatively short mental tests such as MMSE or MoCA will be more suitable for the majority of such patients.

## Diagnosis of POCD

Cognitive function can be evaluated through scales associated with intelligence, memory, cognition and others, clinical manifestations, activities of daily living (ADLs), subjective cognitive concern, NPT batteries, and computer-based cognitive evaluation systems. The subjective cognitive complaint is more sensitive to the patient with richer cognitive reserves. Evaluative tools are the core of diagnosing POCD, and the timing choice of evaluation is also an important part of it. If these tools are conducted too early, it could confuse POCD with POD. However, there is no uniform and recognized timing. As is shown in related researches, the baseline test is usually carried out 1 week before the operation. The early post-operative cognitive test is mostly performed 7 days after the operation. Moreover, the medium- and long-term post-operative cognitive tests often take place at 3 and 12 months after surgery. At this stage, evaluating POCD based on NPT is still the mainstream.

### Z-Score Method

The calculative method for the Z-score of a single test is to subtract from the follow-up score the baseline test score and the mean change on test in the control group, then divide the result by the SD of the change in the control group. The approach yields a combined Z-score for each subject by dividing the sum of Z-scores of all subsets of one person by the SD of the total Z-score in the control group. POCD is defined as at least two Z-scores in individual tests or the combined Z-score is 1.96 or more (Zhang et al., 2018). Some studies also take 2 as the cutoff Z-score, and the diagnosing criterion of POCD is that the combined score, or the Z-scores of at least two subsets, or 20% of the individual NPTs are 2 or more (Rudolph et al., 2008; Rodriguez et al., 2010). The Z-score method usually needs to recruit the control group coincident with the same inclusion/exclusion criteria. Some researchers have proposed a different method without setting a control group, which is just replacing the average and SD of the traditional Z-score with the average and SD of the experimental group. The combined Z-score is the average of all test Z-scores for a single subject, and a negative combined Z-value means a decline in cognitive status (Yocum et al., 2009).

### SD Method

The difference of a single test between the post-operative and preoperative scores is compared with the SD of the baseline scores of the corresponding test. POCD is defined as the changing scores of no less than one test of the testing battery, which are one SD or more (Royse et al., 2011; Klinger et al., 2019). At the same time, exceeding 1 SD is regarded as mild cognitive decline, 1.5 SDs is the moderate cognitive decline and 2 >SDs are severe cognitive decline. Due to the lack of the non-anesthetic/surgical control group, it is impossible to analyze the impact of learning effects and physical cognitive decline caused by aging on NPTs. The SD represents individual differences that are easily affected by multiple factors. If individual variability is great, the false-negative rate will increase, otherwise, the false-positive rate could be high.

### Percentage Method

The percentage method is similar to the SD method. The percentage of the difference between baseline and follow-up scores in the corresponding test is taken as the criterion for POCD. Because there is no control group and no unified standard for the percentage, the reliability of this definition is not satisfying. With the emergence of some new and recognized methods, the frequency of this diagnosing method gradually decreases, and it is not used as the main judging criterion of POCD anymore.

After POCD is integrated into PNDs, the diagnosis of POCD is based on subjective cognitive concern, objective NPTs, and ADLs. At the same time, in terms of NPTs, the difference between baseline and follow-up tests is no longer the compared index. Researchers just need to conduct the post-operative tests and compare them to normal data, which is more comprehensive and in line with the clinical diagnostic criteria. But additional research needs to be conducted to obtain normative reference values that match the different levels of demographic factors at the same time, such as gender, age, and education level.

## Characteristics and Limitations of NPT

The three crucial parts of cognitive-function evaluation are objectively neuropsychological measurement, self-cognitive concern, and a cognitive concern of an informant. NPT belongs to objective assessment, and is not only the main evaluating method of POCD, but is also widely used in the evaluation of cognitive impairment caused by AD, vascular dementia, or Parkinson's disease. An ideal NPT should meet some requirements, such as high sensitivity, specificity, test–retest reliability, and inter-tester reliability. In addition, the overall evaluative process should be simple and easy as well as take a short period of time. Other requirements for an ideal NPT include patients with good compliance, the NPT has alternative versions or a low learning effect, the presence of normative data of this test, the NPT is sensitive to mild changes of cognitive function, the test has corresponding versions of different kinds of languages, and the NPT should not be easily affected by other non-cognitive factors.

With the development of clinical research, more and more attention is paid to limitations of NPT: (1) The diagnosis of POCD requires repeated evaluations of cognitive function and some NPTs, without alternative versions can cause learning effects in repeated tests. This can cover up cognitive impairment or even lead to post-operative cognitive improvement. (2) NPT is prone to a ceiling effect in people with rich cognitive reserves, which reduces its sensitivity. (3) NPT requires professionals to perform in a standard test environment and interpret the test results, which has limitations in clinical applications. (4) A more comprehensive evaluation of the cognitive domains often requires joint application of multiple tests to cover as many cognitive domains as possible. In addition, the time-consuming evaluation process can reduce the compliance of the subject and performer so that the test result cannot reflect the true level of cognition. (5) Different NPTs can cover different cognitive domains. The joint implementation of different NPTs evaluates multiple cognitive domains, but a test does not only involve one cognitive domain so that there will be cognitive overlap among different tests. (6) NPT is easily affected by culture, education level, language, and so on. (7) At present, the normal data of a variety of NPTs are not enough, and the existing data mostly come from smaller clinical samples. (8) The traditional evaluative process has some of the problems of ecological validity, and particular areas are underrepresented, especially those involving high-level cognitive skills and social skills.

## Performance Validity Tests (PVTs)

The NPT score is the main indicator to reflect the cognitive function of the subject. However, the testing scores produced by the subjects in the standard process may not truly represent the real cognitive function and may be influenced by several factors, most notably inadequate task engagement. Invalid results may affect the judgment of cognitive function of the examinee, which can cause some serious repercussions. The national neuropsychological organizations (Bush et al., 2005; Heilbronner et al., 2009) advocated the inclusion of PVTs in routine NPTs many years ago. PVTs have independent and embedded versions. The independent version is still used as the diagnostic criteria for performance validity and includes Medical Symptom Validity Test (MSVT), Test of Memory Malingering-Trail I (TOMM TI), Advanced Clinical Solutions Word Choice Test (WCT), and Dot Counting Test (DCT), etc. The diagnostic indicators of the embedded version are derived from existing NPTs, so it has the dual role of reflecting the cognitive function and test validity. Embedded PVTs are becoming more and more popular in clinical and research environments.

## Outlook

Neuropsychological testing is the main evaluation method of POCD. The integration of traditional neuropsychological evaluation with neurophysiological indicators of EEG or imaging examination can further study the relationship between the human brain and behavior (Marcopulos and Łojek, 2019). Demographic characteristics will affect the validity of NPTs, and this influence can be reduced by establishing norms corresponding to different characteristic levels. With the development of society, digital evaluation tools have attracted more and more attention. Compared with the traditional tools, they have the advantages of high efficiency, accurate result recording, convenient extraction of test results, and less influence by examiners. However, digital tools are the lock of the norms, which may limit its clinical application to a certain extent. Elderly patients have low acceptance of digital testing and lack independent test guidance and supervision, and these factors will also affect the widespread use of digital assessment tools.

Modern evaluation tools will gradually become the assessing mainstream, but the important status of classic evaluation methods still cannot be ignored. The corresponding norms of different tests are not enough, and testing tools are not systematic, which could lead to many inconveniences in mutual quoting. In the future, it will be valuable that there can be more research on these domains, and professionals can establish a global neuropsychological database combined with artificial intelligence. At the same time, with the development of science and technology, the application of new and highly specific digital evaluation methods can make various research or clinical applications more convenient and efficient, and will also obtain more accurate evaluation results.

## Author Contributions

JL, KH, BZhu, BZhou, LL, and XW: equal contribution for the literature search, writing, and correcting of this review article. All authors contributed to the article and approved the submitted version.

## Conflict of Interest

The authors declare that the research was conducted in the absence of any commercial or financial relationships that could be construed as a potential conflict of interest.
